# Mode manipulation in a ring–core fiber for OAM monitoring and conversion

**DOI:** 10.1515/nanoph-2022-0493

**Published:** 2022-11-14

**Authors:** Guowei Wu, Shecheng Gao, Jiajing Tu, Lei Shen, Yuanhua Feng, Qi Sui, Weiping Liu, Zhaohui Li

**Affiliations:** Department of Electronic Engineering, College of Information Science and Technology, Jinan University, Guangzhou 510632, China; State Key Laboratory of Optical Fiber and Cable Manufacture Technology, Yangtze Optical Fiber and Cable Joint Stock Limited Company, Wuhan 430073, China; Southern Marine Science and Engineering Guangdong Laboratory (Zhuhai), Zhuhai 519000, China; Key Laboratory of Optoelectronic Materials and Technologies, School of Electrical and Information Technology, Sun Yat-sen University, Guangzhou 510275, China

**Keywords:** long-period fiber grating, mode filter, mode manipulation, ring-core fiber

## Abstract

The monitoring and conversion of photonic orbital angular momentum (OAM) play fundamental and important roles for both classic and quantum technologies, especially in low-loss transmission media such as ring-core fibers (RCFs), which make many OAM applications practical or vastly more flexible. However, in a RCF, the modes associated with different OAM states are highly overlapping due to the circular refractive index distribution structure, which makes it difficult to distinguish and monitor the OAM modes and in turn limits its inline conversion. Here, we report the first experimental realization of mode monitoring in a RCF using mode filters (MFs), which takes advantage of the difference in the mode adiabatic evolution and the higher-order mode cutoff conditions in tapered RCFs. Different-order OAM can be filtered using MFs with different geometric parameters, as demonstrated by the linearly polarized mode intensity. Combined the mode manipulations in RCF and single-mode fiber, the fundamental mode coupling efficiency can reach 90%, the RCF mode conversion monitoring through inline transmission spectrum evolution can be realized, and the inline fabrication of RCF grating, which couples one mode to a desired mode, can be demonstrated by the fabricating process of three long-period fiber gratings. The mode conversion efficiency between 0-order and 1, 2- or 3-order OAM modes exceeds 96%. Our work provides an efficient approach to monitor and convert OAM modes in higher-order mode supporting RCFs and even other special fibers and further promotes the improvement of the capacity of OAM transmission in RCFs.

## 1 Introduction

Photonic orbital angular momentum (OAM), as a freedom of light found three decades ago [1], has been intensively and extensively investigated through various applications [2–4] concerned with free space, bulk media or waveguides (including fibers). However, in conventional multi-mode fibers (MMFs), the OAM states do not maintain their uncontrollable mix derived from the fiber bend and twist perturbation [5]. Ring-core fiber (RCF) with an annular index profile can address this problem and was found in 2009 [6]. Since that demonstration, heterogeneous RCF design and fabrication have been reported for OAM mode propagation [7–10] and enable a large ensemble of independent OAM mode channels in fibers, and they have been used in both classic [11, 12] and quantum communication links [13, 14]. Benefitting from similar profiles with OAM modes and unique mode-coupling characteristics, low differential mode gain (DMG) amplifiers and low digital signal processing complexity are realized in RCF systems [15], which can further promote OAM applications. In RCF optics communication, due to the unique structure of RCF and the excellent characteristics of OAM modes, photons encoded in OAM space can significantly increase the information channel capacity and transmission distance [16–19], such as in a 1 Pbps OAM mode multiplexing transmission system [20]. With the further improvement of OAM multiplexing transmission in RCFs, mode monitoring and conversion are needed for the full utilization of the OAM space or the spatial bandwidth source. Beyond fiber-optic communications, mode monitoring or recognition has also seen applications in versatile scenarios such as sensing, nonlinear optics, mode conversion and classic and quantum networks [21–24]. To date, such a mode monitoring scheme is further used for mode conversion, which remains difficult due to the high overlap of OAM modes in RCFs.

There are some experimental realizations of mode recognition or monitoring, either by using a deep learning algorithm or by inline mode conversion [25]. Adiabatic mode evolution employing filtering arbitrary modes is much more attractive for practical applications because it simply measures the power or transmission spectrum of the input and output ports to deduce the mode components, in contrast to using the above methods. Most previous works have demonstrated adiabatic fundamental mode evolution either in MMFs or in multicore fibers [26–28]. However, for the higher-order modes, the adiabatic taper criterion in these fibers, especially RCF, is more challenging because the mode field diameter (MFD) changes rapidly when the mode is cutoff from the core. Although T. A. BIRKS et al. [29, 30] proposed and demonstrated a fiber with a logarithmic refractive index profile that can realize the fundamental mode and higher-order mode transmission with low loss for endlessly adiabatic purposes, it is difficult to employ common optical fibers (e.g., step-index or graded index fibers). To simultaneously achieve higher-order mode cutoff and other mode adiabatic evolution, precisely controlling the geometric parameters and refractive index profile of RCF is needed, which determines the number of modes and all the key optical properties. Typically, the RCF taper is formed by quickly locally heating a fiber above the softening temperature of the glass while simultaneously stretching the fiber so that the softened material narrows down; thus, the refractive index profile remains almost unchanged, and the geometric parameters, including waist diameter and transition zone length, become the only factors. The waist diameter determines the number of modes, that is, the higher-order mode cutoff. The transition zone length follows the “weak power transfer” criterion of Love et al. [31], which must be longer than the maximum beat length between two arbitrary modes because the mode field distribution changes along the fiber [32]. In this case, adiabatic mode evolution with a higher-order mode cutoff could be realized and used to distinguish the modes in RCF with highly overlapping and further promote mode conversion in RCFs.

Mode conversion in RCF offers a desired mode to be multiplexed for mode-division multiplexing systems reduces additional loss and enhances compatibility; hence, there is an increasing interest in mode conversion in RCFs. To realize mode conversion between different-order OAM beams, various approaches, including cascaded four-mode long-period fiber grating (LPFG) pairs [33], helical LPFG [34] and tilted few-mode fiber Bragg grating [35], have been reported and demonstrated. However, it is difficult to complete the inline fabrication of these fiber devices while applying them to RCF. In RCFs, a mode-selective coupler [36], pressing combined with tapering method [37] and acoustic-induced LPFG [38] have been proposed to realize mode conversion. However, their manufacturing process is complicated and unstable, and they can only realize a low-order mode conversion. The Ref. [37] realized the first-order OAM mode generation by tapering and pressing methods, but the low coupling efficiency of the first-order OAM mode and difficulty in higher-order OAM mode generation remains a problem. As one of the most promising candidates, LPFG has the merits of simple structure, flexible operation, low loss and low crosstalk. Li et al. [39] proposed an OAM mode conversion scheme in RCF, and TE_01_, TM_01_ and HE_11_ have been generated in theory. LPFG in experimental implementation has not been reported until now.

In this paper, we propose and demonstrate a mode recognition and monitoring scheme using mode filters (MFs), which take advantage of the difference in the mode adiabatic evolution conditions and the higher-order mode cutoff in tapered RCFs. Different-order OAM modes can be filtered using MFs with different geometric parameters, as demonstrated by the linearly polarized (LP) mode intensity. Furthermore, combined with the mode manipulations in RCF and SMF, the RCF mode conversion monitor by inline transmission spectrum evolution has been realized, and the inline fabrication of RCF grating, which couples one mode to the desired mode, has been verified by the fabrication process of three LPFGs. The fundamental mode efficiency between RCF and SMF can reach 90% by the transmission spectrum and interference diagram. The mode conversion efficiency between the 0-order and 1-, 2- or 3-order OAM modes exceeds 96%. Our study provides an efficient mode manipulation scheme for OAM monitoring and conversion in higher-order mode supporting RCFs and even other special fibers.

## 2 Principle

### 2.1 Mode analysis in RCF

**Figure 1:**
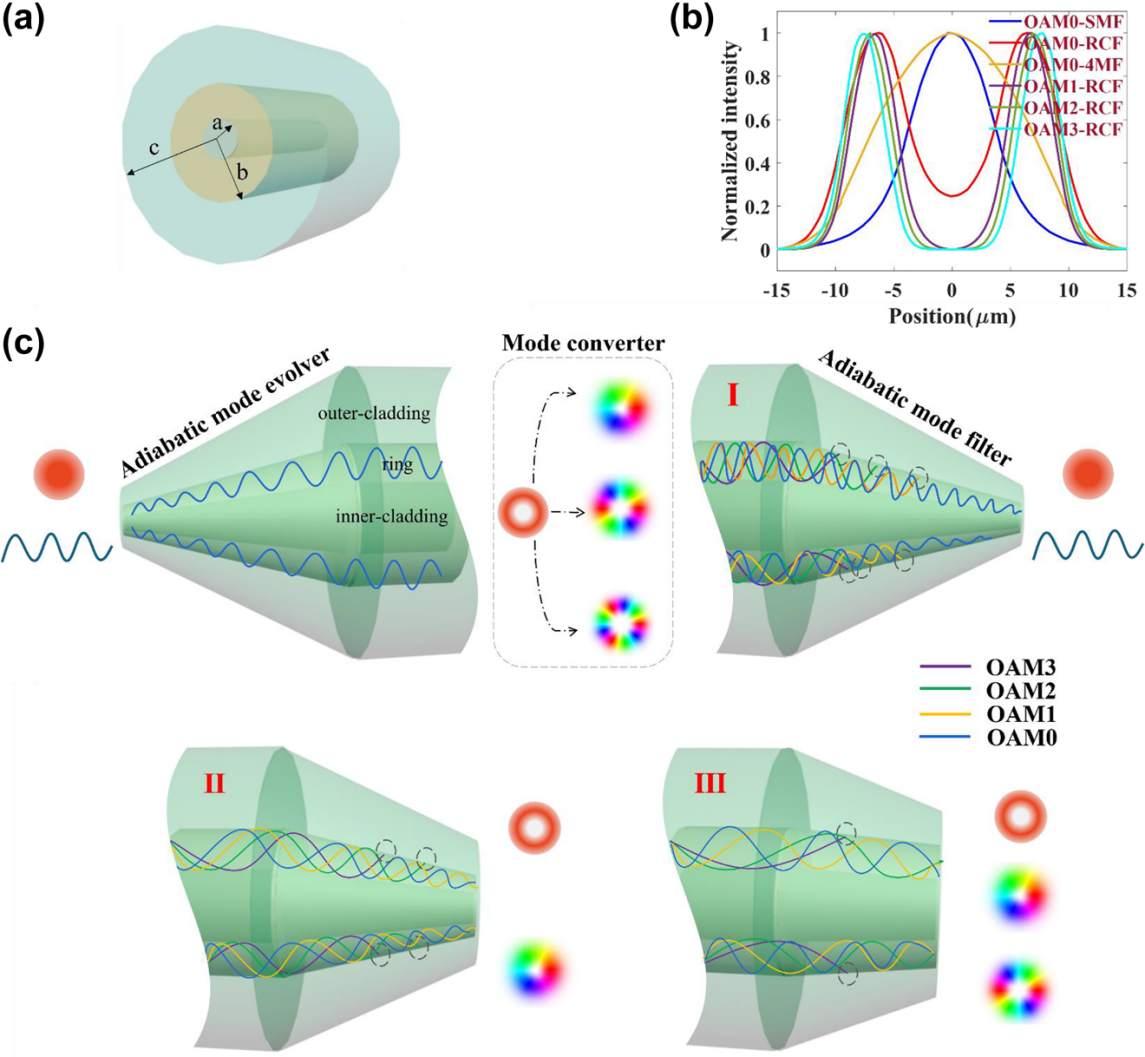
The schematic diagram. (a) Schematic of the ring-core fiber. (b) The normalized intensity profile of the fundamental mode in SMF, 4 MF and each order mode in RCF. (c) Schematic of MFs.

To manipulate the modes in RCF, we first need to know the mode profile in the fiber. A schematic of the RCF is shown in Figure 1(a). The mode profile in the RCF, namely, three layers of fiber structure, can be expressed as follows [40]:

(1) $$\Psi_{l}\left(r,\varphi \right)=S\left(l\varphi \right)\left\{\begin{array}{c} C_{1}I_{l}\left(wr\right)\;\\ A_{1}J_{l}\left(ur\right)+A_{2}Y_{l}\left(ur\right)\;\\ C_{2}K_{l}\left(wr\right)+C_{3}I_{l}\left(wr\right)\; \end{array}\right.\;\begin{array}{c} 0\leq r<a\\ \;a\leq r<b,\\ b\leq r\leq c \end{array}$$

where *S*(*lφ*) is either cos(*lφ*) or sin(*lφ*) depending on the mode parity, where *J*_*l*_ and *Y*_*l*_ are the Bessel functions of the first and second kind, *K*_*l*_ and *I*_*l*_ are the modified Bessel functions of the first and second kind and *a*, *b* and *c* are the radii of the inner and outer annular ring and cladding, respectively, as shown in Figure 1(a). Real coefficients *A*_*i*_ and *C*_*i*_ are determined by the condition that fields described by Eq. (1) must be continuous and smooth across all the fiber sections and null in the cladding border *r* = *c* to solve the scalar wave equation. From Eq. (1), the mode profile in the RCF is not similar to that in the SMF or few-mode fiber (FMF) (four-mode fiber (4 MF) as an example). Meanwhile, the fundamental mode profile in RCF presents a ring shape, and the overlap between it and SMF is quite low, as shown in Figure 1(b) and calculated at wavelength of 1550 nm (all the places not mentioned) by COMSOL. Therefore, it will cause a large loss and other modes excitation if the RCF is directly spliced with the SMF or FMF.

A variety of reports [41, 42] have provided reverse taper and thermal expand core methods to improve the fundamental mode efficiency in FMFs or MMFs during splicing with a SMF, which is not suitable for RCFs because of their unique annular core. Ref. [37] proposes a symmetric taper method between fused SMF and RCF, the separate mode manipulation in each fiber, which is beneficial to realize a desired mode coupling or monitoring, and higher-order mode cutoff is still an issue. For this reason, we propose a MFs scheme, and the schematic diagram is shown in Figure 1(c). As a proof-of-concept, the RCF can support four OAM mode groups. The input fundamental mode of the adiabatic mode evolver can be converted to the mode in RCF with low loss, and a higher-order mode cannot be excited in this process because one can strictly control the geometric parameters and ensure the higher-order mode cutoff and fundamental mode adiabatic evolution. After that, the fundamental mode in RCF can be converted to a desired higher-order OAM mode by a mode converter, and these modes cannot pass the adiabatic mode filter, which is the same as the adiabatic mode evolver before the mode converter. The fundamental mode in RCF can successfully pass the mode filter, and then this structure can act as a mode conversion monitor. Moreover, the two different MFs (smaller taper angle) satisfying higher-order adiabatic conditions are shown in the last row of Figure 1(c), and the 3-order mode or 2-order and 3-order mode can be filtered. This method can be applied to other fibers. Therefore, it is possible to achieve mode manipulation in RCF for OAM monitoring and promote the fabrication of LPFG employing the MF.

### 2.2 Mode manipulation in RCF

**Figure 2:**
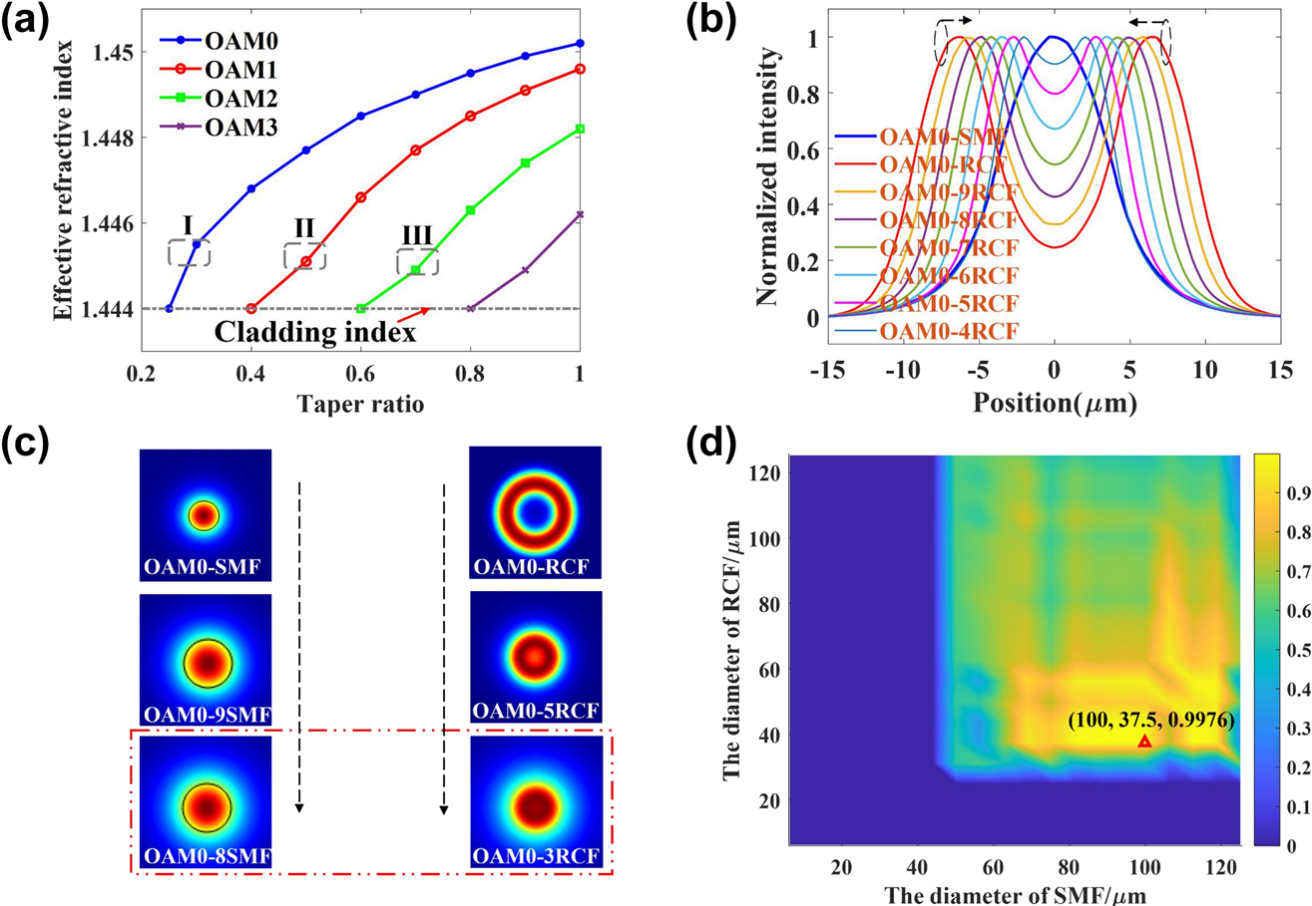
The simulation results. (a) The refractive index variation of each order mode in the tapered RCF. (b) The normalized intensity profile of the fundamental mode in SMF and (tapered) RCF. (c) Fundamental mode evolution in two tapered fibers. (d) The relationship between the fundamental mode coupling efficiency and the diameter of the SMF and RCF.

To further investigate the feasibility of the scheme, mode monitoring is performed using MFs. First, as shown in Figure 2(a), we calculate the relationship between the effective refractive index of the OAM modes supported by the RCF and the taper ratio, which means that the diameter of the waist divides into the original diameter of 125 μm. When the effective refractive index of one mode is lower than the refractive index of the cladding, the mode is cutoff in the waist zone. Thus, as the diameter of the waist decreases, the higher-order OAM mode gradually disappears. That means that we can make other higher-order OAM modes cutoff and monitor whether one mode exists in this fiber only by strictly controlling the geometric parameters that the adiabatic conditions can be meeting. As a proof, I, II and III have been selected to verify the feasibility, as shown in Figure 2(a), which corresponds to Figure 1(c). The other region that ensures the other modes cutoff is also acceptable, which means our approach has great tolerance.

Second, the fundamental mode coupling efficiency between the tapered RCF and SMF is also studied for the transmission spectrum inline monitoring of a LPFG in the fabrication process. As shown in Figure (b) and (c), the fundamental mode profile would be more likely with the diameter of two fibers decreasing and mode fields correcting. The *x*RCF or *x*SMF means that the diameter of the tapered fiber is 0.1× *x* times the original diameter. From a quantitative point of view, the relationship between the fundamental mode coupling efficiency and the diameter of the RCF and SMF have been calculated as shown in Figure 2(d), and the coupling efficiency expression can be expressed as Eq. (2). In Eq. (2), *E*_1_ and *E*_2_ represent the electric field distribution of RCF and SMF, and $E_{1}^{*}$ and $E_{2}^{*}$ represent the conjugate of *E*_1_ and *E*_2_. The fundamental mode is cutoff in the blue zone, and the highest coupling efficiency is 99.76% when the diameters of RCF and SMF are 37.5 μm and 100.0 μm, respectively. Therefore, fundamental mode coupling with high efficiency between these two mismatched fibers can be realized by combining the MF and tapered SMF.

(2) $$\eta =\frac{\iint E_{1}E_{2}^{*}dxdy}{\sqrt{\left|\iint E_{1}E_{1}^{*}dxdy\right|\cdot \left|\iint E_{2}E_{2}^{*}dxdy\right|}},$$

**Figure 3:**
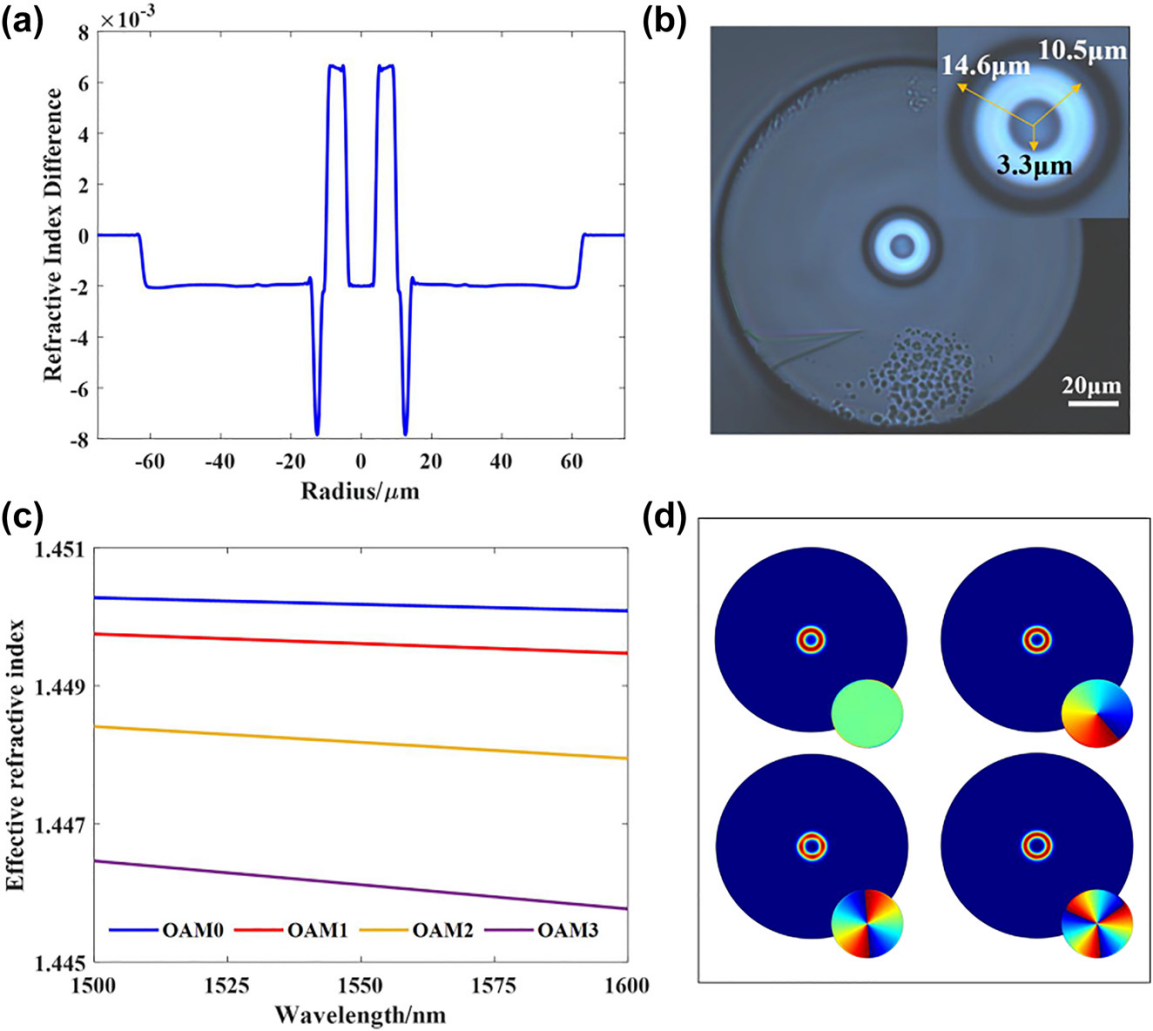
The relative parameters of ring-core fiber. (a) The refractive index profile; (b) the end face image of RCF; (c) the dispersion curve of RCF; (d) OAM modes profile supported by the RCF.

In this letter, the optical fiber was RCF with a step-index profile. The refractive index profile is shown in Figure 3(a), and the fiber end face was observed by microscopy and recorded in Figure 3(b). The refractive index difference between the annular guide layer and cladding is approximately 0.009, and the radii of the inner and outer annular layer and trench layer are approximately 3.3 μm, 10.5 μm and 14.6 μm, respectively. By using COMSOL, the effective refractive index of all modes in the wavelength range of 1500 nm 1600 nm was calculated and recorded, as shown in Figure 3(c). The RCF can support the transmission of OAM_0_, OAM_±1_, OAM_±2_ and OAM_±3,_ which is shown in Figure 3(d).

**Figure 4:**
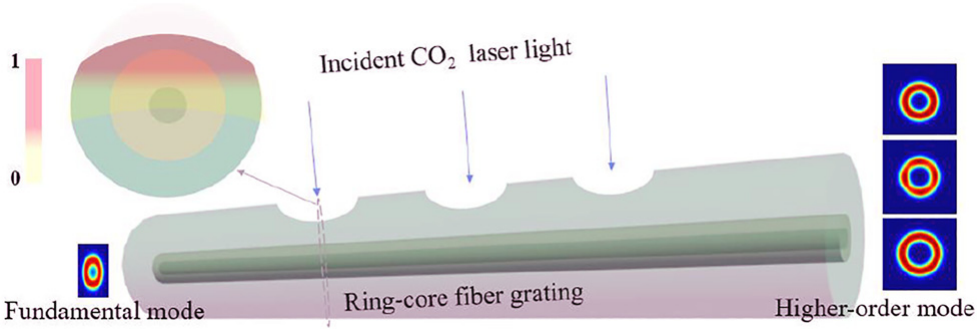
Schematic diagram of LPFG inscribed in the RCF.

**Figure 5:**
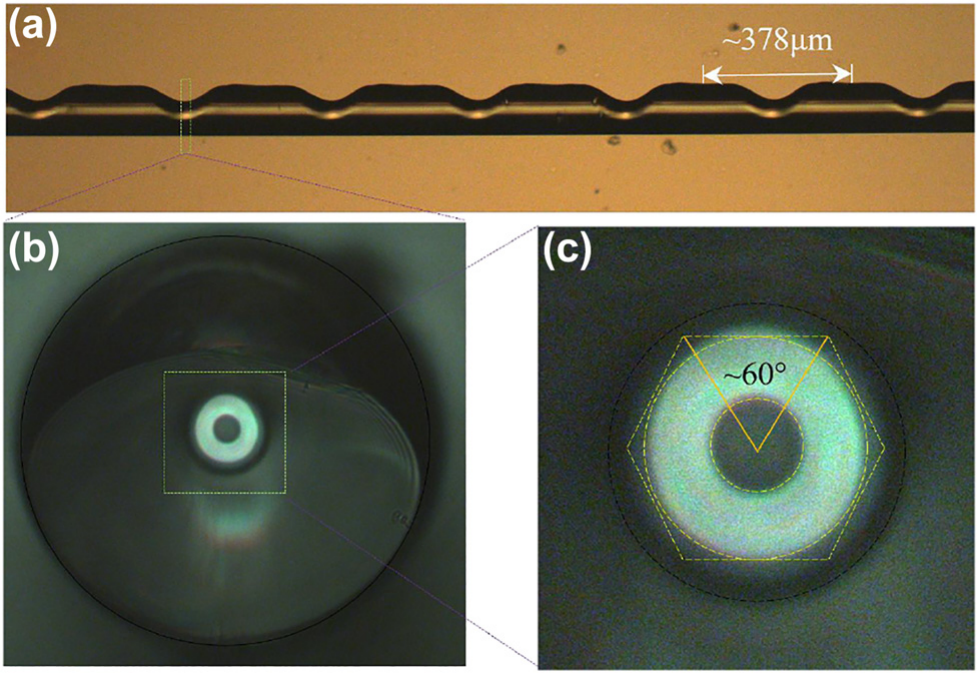
Images of the 3-order RCF grating. (a) Side view of the 3-order RCF grating; (b) and (c) end face of the 3-order RCF grating.

In addition, as shown in Figure 4, the fundamental mode is coupled to a desired higher-order OAM mode by passing the periodic refractive index modulation LPFG, and the cross-section of the modulation zone is shown in the insert figure of Figure 4. According to the phase matching condition of Eq. (3), the grating periods of the mode conversion between the 0-order and 1-, 2- or 3-order OAM modes are 2.727 mm, 0.7754 mm and 0.3817 mm at 1550 nm, respectively. In Eq. (3), *n*_*f*_ and *n*_*h*_ represent the effective refractive index of the fundamental mode and higher-order OAM mode, respectively, Λ is the grating period and *λ* is the resonance wavelength. The processing of LPFG uses a CO_2_ laser (Han’s 30) with scanning speed of 27 mm/s. As an example, Figure 5 shows the images of the 3-order grating. The exposure power of CO_2_ laser is three times for 2.7 W, once for 3.3 W, once for 3.75 W and once for 4.35 W. The period of this grating is approximately 378 μm, which is basically consistent with the result of theoretical calculations, and the deformation angle of the fiber is approximately 60°, which corresponds to the angle of each lobe of LP_31_.

(3) $$\Lambda =\frac{\lambda }{n_{f}-n_{h}},$$

## 3 Experimental results

### 3.1 Mode filters for mode monitoring

**Figure 6:**
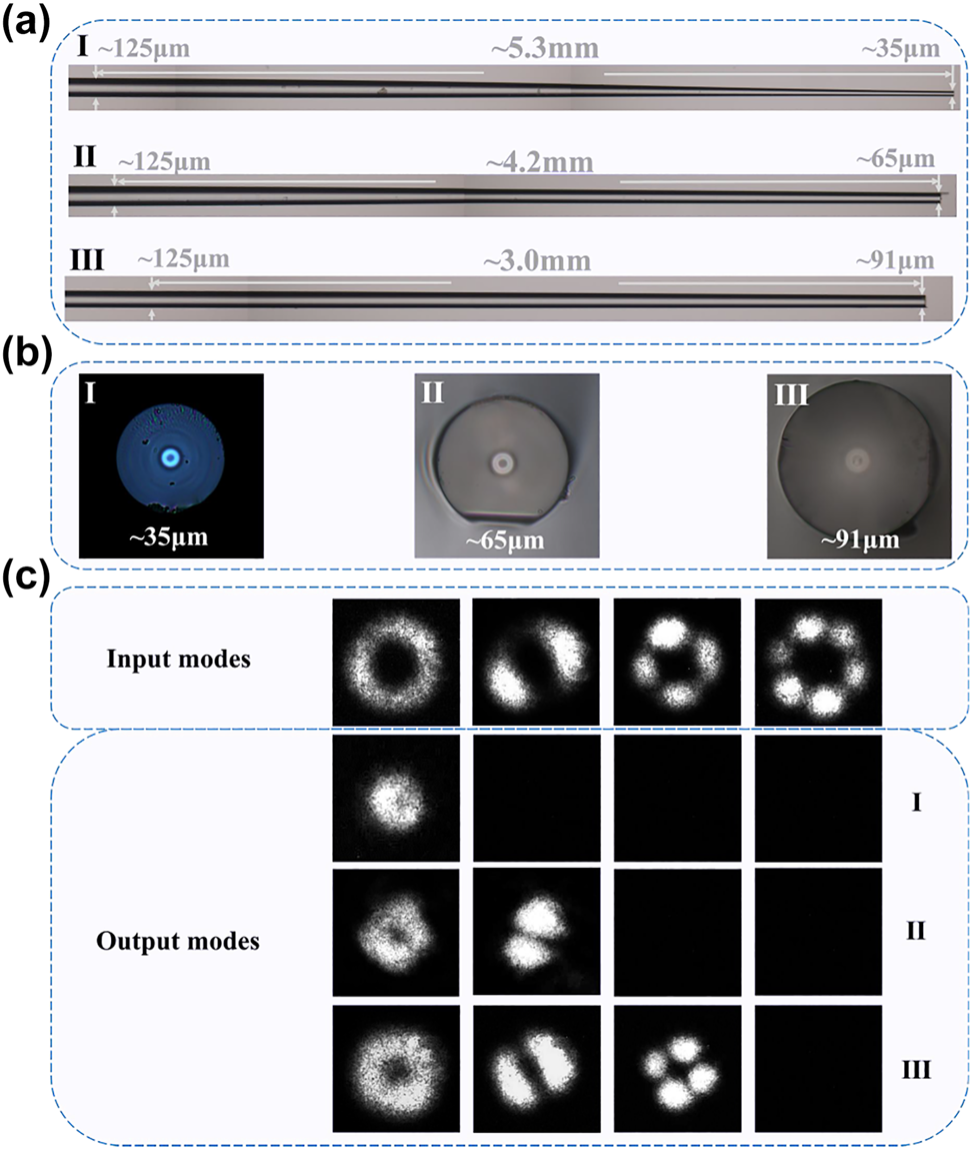
The experimental results of MFs. (a) The side view, (b) end face and (c) mode evolution of three MFs.

To fabricate the MF and further demonstrate the experimental effect of mode manipulation for mode monitoring. First, three MFs with different geometric parameters for mode monitoring were fabricated by using a hydrogen oxide flame. The side view and the end face of these MFs were observed and recorded in Figure 6(a) and (b). The diameters of the taper waist of the three MFs are ∼35 μm, ∼65 μm and ∼91 μm, which correspond to Figures 1(c) and 2(a). The output mode profiles of these MFs at 1550 nm are shown in Figure 6(c) under each order LP mode supported by the fiber as the input mode which is input from the left of Figure 6(a). Because the fiber cannot support one mode in the diameter of waist of the MF, the output mode profile is none. Furthermore, the intensity is larger than that of the input mode, and the mode distribution range is smaller or more restricted than that of the input mode, which means that higher-order mode transmission with low-loss or adiabatic conditions is met.

**Figure 7:**
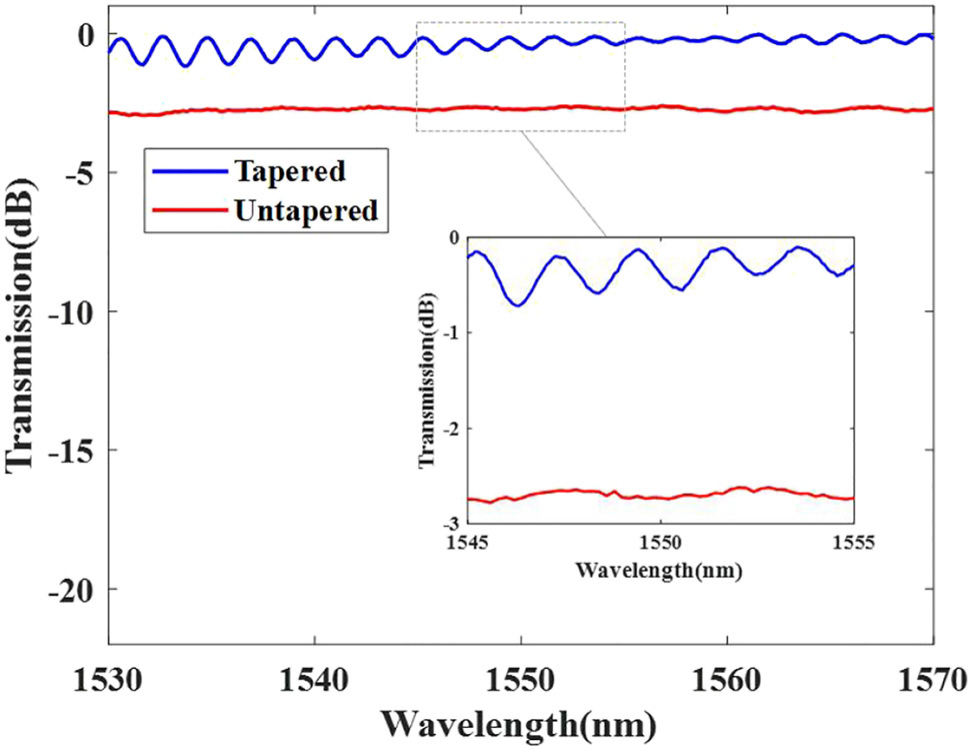
Transmission loss of the tapered and untapered fibers.

Moreover, by tapering the SMF and the diameter of its waist to 100.0 μm and combining with the MF, whose diameter is 37.5 μm, with all higher-order modes cutoff, the sample was successfully fabricated, as shown in the insert figure of Figure 10, and can be used to inline monitor the transmission spectrum evolution. The sample is carefully spliced with the tapered SMF using Furukawa S178A. Figure 7 shows the transmission loss before and after processing, and the tapered and untapered curves represent the sample and the result of directly splicing the SMF with RCF. The energy coupling efficiency has been largely enhanced, and the transmission loss is approximately 0.5 dB and even 0.1 dB at some wavelengths employing this method.

**Figure 8:**
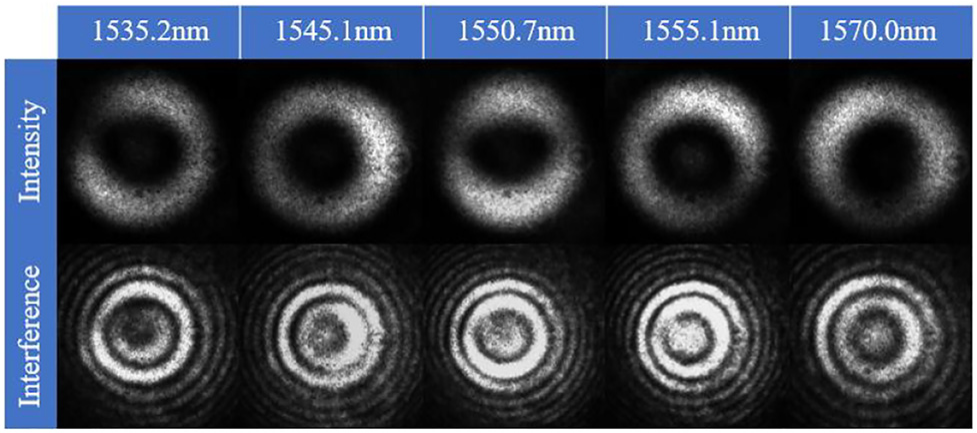
The intensity and interference diagram of output modes of the sample.

To further describe the transmission mode in the RCF as the fundamental mode, an interference experiment whose setup is shown in Figure 10 was designed, and the results are shown in Figure 8. Five wavelengths that represent the larger and smaller losses have been selected for verification, and the intensity and interference diagram are fundamental modes. This means that we can realize mode manipulation for OAM monitoring using this method, and the sample that achieves fundamental mode transmission with almost no loss can be used to inline monitor the transmission spectrum evolution during the fabrication of the LPFG.

### 3.2 Long-period fiber grating for mode conversion

**Figure 9:**
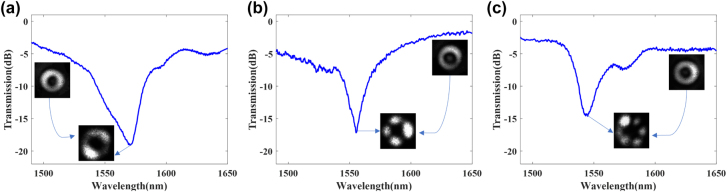
Experimental transmission spectrum of RCF grating for (a) the 1-order, (b) the 2-order and (c) the 3-order.

To demonstrate the mode monitoring and manipulation for mode conversion experimentally, three LPFGs were fabricated by using a CO_2_ laser. First, the transmission spectrum of these LPFGs was monitored and recorded, as shown in Figure 9. SMF was connected with the supercontinuum broadband light source (Fianium, WL-MICRO) and an optical spectrum analyzer (OSA, Yokogawa, AQ6370D) at both ends and two MFs were used to connect to the RCF at the middle of SMF. The transmission spectrum is straight and stable before writing, and the resonance dip at one wavelength appears when resonance coupling occurs, which means that the fundamental mode is coupled into a higher-order OAM mode. Figure 9 shows the three LPFGs according to the mode conversion between the 0-order and 1-, 2- or 3-order OAM modes at 1570.0 nm, 1555.1 nm and 1545.2 nm. The period numbers of these LPFGs are 12, 60 and 86. The periods are 3 mm, 0.86 mm and 0.378 mm. The mode conversion efficiencies are 98.7%, 98.0% and 96.7% and the insertion loss are about −3.2 dB, −1.7 dB and −2.9 dB, respectively.

**Figure 10:**
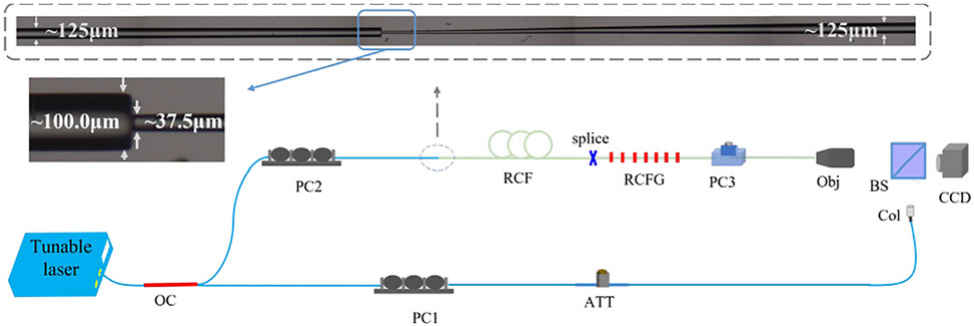
Experimental setup for the mode conversion and detection. OC, 1:1 optical coupler; PC, polarization controller; Obj, objective; ATT, attenuator; BS, beam splitter; Col, collimator; RCFG, RCF grating; CCD, charge-coupled device.

Furthermore, to further illustrate that the converted modes are the designed and desired mode, a system, as shown in Figure 10, was constructed to record the higher-order OAM mode and its interference diagram. A tunable laser can generate fundamental Gaussian light in the C + L band, which is divided into two paths by a 1:1 optical coupler (OC). The upper path can generate the higher-order OAM mode by a series of optical devices. PC2 can adjust the polarization state of the input light, and PC3 is used to form an OAM mode by adjusting the phase and polarization of the $LP_{l1}^{\mathrm{even}}$ and $LP_{l1}^{\mathrm{odd}}$ modes generated by the RCF grating which can generate first-, second- or third-order modes. The sample shown in the insert figure of Figure 10 guarantees that the output mode of RCF is the fundamental mode, which has been verified in Section 3.1. The length of the transition zone of two tapered fibers is about 3.0 mm and 5.0 mm. The down road is used as a Gaussian reference light, whose length is matched to the length of the upper path. The beam splitter (BS) can combine two beams of upper and lower paths to form an interference diagram recorded by the charge-coupled device (CCD).

**Figure 11:**
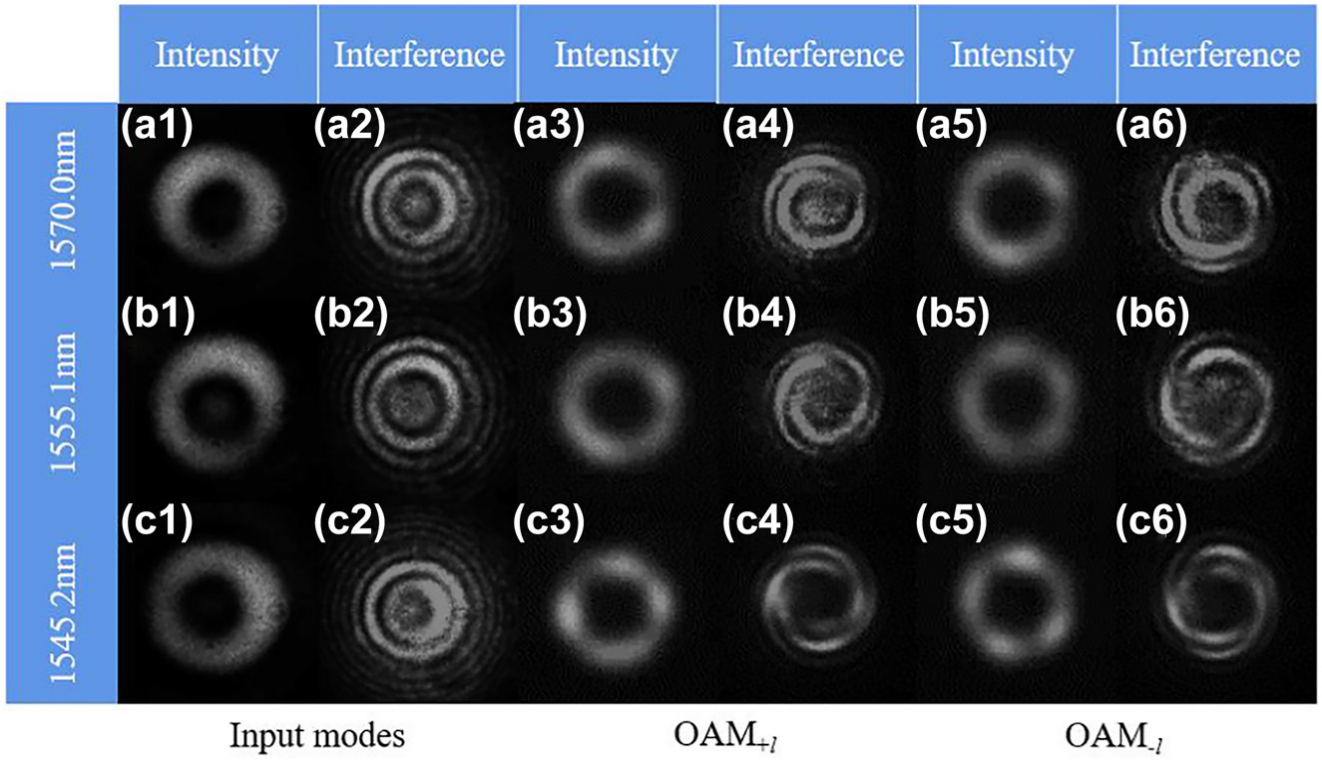
The input and output OAM mode profiles and interferences with Gaussian beam for (a) the 1-order, (b) the 2-order and (c) the 3-order.

The mode intensity and interference diagram before and after RCF gratings at their resonance wavelength are recorded in Figure 11(a)–(c) represent the results of first-, second- and third-order RCF grating at their resonance coupling wavelengths, which is shown in Figure 9(a)–(c). The first two columns represent the intensity and interference diagram of the input mode of the RCF grating, which means that the input modes are the fundamental mode with a ring shape. The middle two columns represent OAM_+*l*_ and the last two columns are OAM_−*l*_. The mode intensity is in the shape of a donut, and spiral fringes appear after interference with Gaussian light. According to these results, the mode conversion between the fundamental mode and higher-order OAM modes reaching the highest-order OAM mode supported by the RCF with mode conversion efficiency greater than 96% can be realized. The mode purity is measured using the method in [43, 44] and the results are more than 85% for three RCF gratings. In addition, according to Eq. (3), the mutual mode conversion between the fundamental mode and the higher-order OAM mode could be performed employing LPFGs. This means that the output mode would be a fundamental mode when the higher-order OAM mode is used as an input mode under the relative RCF grating. Therefore, in general, mode manipulation for OAM conversion can be realized under the concrete OAM mode as the input mode.

## 4 Conclusions

In summary, a mode manipulation scheme for mode monitoring and conversion based on MFs and LPFGs has been proposed and demonstrated experimentally. The MF can be used as an adiabatic mode evolver or monitor, which realizes the desired higher-order mode cutoff and other OAM modes adiabatic transmission in RCFs. Furthermore, combined with the MF and tapered SMF, the fundamental mode coupling between the two fibers can be enhanced to 90%, and the sample can also be used to monitor the transmission spectrum evolution in the process of inline fabrication of RCF gratings. Three mode converters of RCF gratings, which realize the mode conversion between 0-order and 1-, 2- or 3-order OAM modes, have been fabricated and are used to demonstrate the mode manipulation for mode conversion. The mode conversion efficiency of these gratings is 96% above. Our study will find a potential application in high-resolution imaging, vortex-optics sensing and mode recognition, and further promote OAM multiplexing transmission in these fibers to improve the transmission capacity and distance.
